# Psychometric properties for Persian version Demoralization Scale‐24 (DS‐24) in Iranian cancer patients

**DOI:** 10.1002/brb3.3589

**Published:** 2024-07-29

**Authors:** Nazanin Mousavi, Mandana Piryaei, Roghieh Nooripour, David Kissane, Zahra Hooshyari, Mohammad Effatpanah, Nikzad Ghanbari

**Affiliations:** ^1^ School of Psychology Imam Khomeini University Qazvin Iran; ^2^ Department of Psychology, Faculty of Literature and Humanities University of Guilan Rasht Iran; ^3^ Department of Counseling Qazvin Branch, Islamic Azad University Qazvin Iran; ^4^ Department of Counseling Faculty of Education and Psychology, Alzahra University Tehran Iran; ^5^ Cunningham Centre for Palliative Care Research, St Vincent’s Sydney and Szalmuk Family Research Unit at Cabrini Health University of Notre Dame Australia Clayton Australia; ^6^ Faculty of Psychology and Education University of Tehran Tehran Iran; ^7^ Professor of child & Adolescent Psychiatry Pediatric Department, school of medicine, Imam Khomeini hospital, Tehran University of Medical Science Tehran Iran; ^8^ Department of Clinical Psychology Faculty of Psychology and Educational Sciences, Shahid Beheshti University Tehran Iran

**Keywords:** cancer, demoralization, patient, translation, validity

## Abstract

**Background:**

This study focuses on the phenomenon of demoralization, a common experience among terminally ill patients, especially those diagnosed with cancer. The primary objective is to adapt and validate a practical assessment tool for demoralization, the Demoralization Scale‐24 (DS‐24), within the context of Iranian society.

**Methods:**

In this cross‐sectional study, we employed the DS‐24 as the principal instrument, which had been exactly translated and evaluated for its psychometric properties in 160 Persian cancer patients. The assessment included exploratory factor analysis, confirmatory factor analysis (CFA), as well as evaluations of convergent validity and internal consistency or reliability.

**Results:**

The CFA unveiled a five‐factor model, consistent with the original structure of the DS‐24. Moreover, statistically significant correlations were observed between the DS‐24 and both the Beck Depression and MUNSH happiness scales. Cronbach's alpha indicated high internal consistency, with a value of .92 for the total score.

**Conclusion:**

In Iran, like in other countries, the demoralization questionnaire demonstrates significant validity and reliability. This ensures the timely diagnosis of demoralization in cancer patients and the prompt initiation of therapeutic interventions.

## Graphical abstract

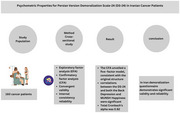

## 1 INTRODUCTION

The mortality rate associated with cancer remains alarmingly high, primarily attributed to the adverse impact of both hereditary factors and environmental influences on an individual's genetic composition (Figures, 2020). As one of the foremost contributors to health‐related challenges, cancer significantly affects individuals within societies worldwide, including a notable burden on their psychological well‐being. A comprehensive report from the Health Organization reveals that cancer ranks as the primary or secondary cause of death among individuals aged 70 and above in 112 out of 183 countries (Sung et al., 2021). Notably, the prevalence of cancer within societies is witnessing an upward trajectory, with predictions indicating an estimated 27,500,000 individuals affected by this disease and 16,300,000 fatalities projected by the year 2040 (Figures, 2020). Various risk factors contribute to the incidence of cancer, notably population growth and aging, which escalate the overall risk. Specific to Iran, prevalent risk factors include substance consumption, such as opium and tobacco, inadequate consumption of fruits and vegetables, sedentary lifestyle habits, and the excessive consumption of hot tea (Sheikh et al., 2019, 2020).

Numerous studies conducted by Mitchell et al. (2011) and Walker et al. (2014) have shed light on the psychological challenges faced by cancer patients. These studies have consistently found high levels of emotional distress and psychological problems among individuals diagnosed with cancer (Mitchell et al., 2011; Walker et al., 2014). Meta‐analytic investigations have further explored the prevalence of psychological disorders in this patient population over a 12‐month period. The findings indicate that the prevalence rates of mood disorders, anxiety disorders, and somatoform disorders are 18%, 19%, and 8%, respectively. Additionally, the lifetime prevalence rates of anxiety disorders, adjustment disorders, mood disorders, and somatic disorders have been reported as 21%, 21%, 26%, and 16%, respectively (Mehnert et al., 2013; Vehling et al., 2012). These findings highlight the substantial burden of psychological disorders experienced by cancer patients.

Demoralization is a notable factor observed among cancer patients and individuals with incurable diseases, with a higher prevalence observed in patients compared to the general community (0.8%–71% vs. 19.8%, respectively) (Clarke & Kissane, 2002; Roberts & Vernon, 1981). Various definitions of demoralization have been proposed in the literature. Frank & Frank (1961) described demoralization as a state of giving up, characterized by feelings of failure or mental incompetence when individuals struggle to fulfill their own desires or encounter difficulties in problem‐solving (Frank & Frank, 1961). Schmale and Engel (1967), on the other hand, defined demoralization as a psychological condition preceding illness, characterized by helplessness and a sense of failure. In the context of incurable patients such as those with cancer, demoralization has been conceptualized as existential distress, characterized by a profound sense of helplessness and hopelessness, leading to a loss of purpose and meaning in life (Kissane et al., 2001). Importantly, demoralization has been associated with a heightened risk of suicide, emphasizing the need for careful attention to this psychological phenomenon (Kissane, 2004). Studies have further revealed that the existential stress arising from demoralization is correlated with physical symptoms in cancer patients (Grassi et al., 2004; Vehling et al., 2013). And also, demoralization is correlated with psychiatric disorders; prior research indicates substantial correlations between demoralization and self‐reported depression as well as self‐reported anxiety (Costantini et al., 2013; Lee et al., 2012). De Figueiredo (1993) highlighted that individuals experiencing depression or demoralization tend to lack motivation and feel inhibited from taking action. People who suffer from demoralization might not find joy and may have a sense of gloominess about the future. Czapinski's (1992) theory posits that happiness comprises multiple layers, with the desire for life and passion for life being the most stable elements. The cessation of life pursuits represents a pivotal aspect for individuals afflicted with demoralization. Thus, it is anticipated that those exhibiting elevated levels of demoralization would manifest reduced levels of happiness (Czapinski, 1992; Strada, 2009).

Several researchers have developed measurement tools to assess demoralization in individuals. Examples of these tools include the Psychiatric Epidemiology Research Interview (Dohrenwend et al., 1980), the Minnesota Multiphasic Personality Inventory, version 2 (Nichols, 2011), the Diagnostic Criteria for Psychosomatic Research (Fava et al., 1995), and the Demoralization Scale (DS). However, among these instruments, the demoralization questionnaire designed by Kissane et al. (2004) has demonstrated notable effectiveness in measuring demoralization, specifically in patients with incurable conditions such as cancer. The DS, developed by Kissane et al. (2004), is a measurement tool specifically designed to assess demoralization. The sample used in the development of the scale consisted of 100 patients with advanced cancer who met the inclusion criteria. Participants completed several other questionnaires alongside the DS, including the McGill Quality of Life, Patient Health Questionnaire, Beck Depression Inventory, Beck Helplessness Scale, Hunter Opinions and Personal Expectation Scale, and Schedule of Attitudes Toward Hastened Death. The DS questionnaire comprises 24 items and measures 5 subscales. Responses are scored on a five‐point Likert scale ranging from 0 (never) to 4 (all the time). The reliability of the questionnaire for each subscale, as assessed by Cronbach's alpha, is as follows: loss of meaning and purpose (five items, *α* = .87, M = 5.12, SD = 4.48), dysphoria (five items, *α* = .85, M = 6.35, SD = 4.49), disheartenment (six items, *α* = .89, M = 8.91, SD = 5.76), helplessness (four items, *α* = .84, M = 5.25, SD = 4.07), and sense of failure (four items, *α* = .71, M = 5.19, SD = 2.88). The questionnaire has also shown acceptable content validity and divergent validity (Kissane et al., 2004).

This questionnaire has been translated into various languages, and its validation has been examined in different populations. For example, Cheng et al. (2019) evaluated the scale in the Chinese breast cancer community and identified four components with a variance of 58.66% using exploratory factor analysis (EFA). Cronbach's alpha for the components ranged from .720 to .894, demonstrating acceptable internal consistency (Cheng et al., 2019). Similarly, Mehnert et al. (2011) evaluated the questionnaire in a German sample and identified four components that accounted for 0.59% of the variance. Cronbach's alpha values were acceptable for each component (loss of meaning and purpose = .88, disheartenment = .88, dysphoria = .80, sense of failure = .76) (Mehnert et al., 2011). In a study involving the Spanish population, Rudilla et al. (2016) reported good fit results from confirmatory factor analysis (CFA), and five components were identified using exploratory structural equation modeling, excluding item 18. Cronbach's alpha values for the components were as follows: loss of meaning and purpose = .86, helplessness = .79, disheartenment = .88, dysphoria = .80, and sense of failure = .62 (Rudilla et al., 2016). Finally, Grassi et al. (2017) examined the scale in an Italian sample and extracted four components accounting for 57.1% of the variance. Cronbach's alpha values were as follows: disheartenment = .87, dysphoria = .73, sense of failure = .77, and loss of meaning and purpose = .72 (Grassi et al., 2017).

The measurement of demoralization in cancer patients holds significant importance, and the demoralization questionnaire developed by Kissane et al. (2004) has proven to be a suitable scale for this purpose. Therefore, the primary objective of this study is to translate and evaluate the validity of the demoralization questionnaire in the context of Iranian society.

## 2 MATERIALS AND METHODS

### 2.1 Participants

The present study adopts a descriptive and cross‐sectional research design. The target population consists of cancer patients seeking treatment at Imam Khomeini Hospital in Tehran between the years 2021 and 2022. Through the utilization of convenience sampling, a sample of 200 patients was selected, and 160 patients completed the questionnaires. Before the implementation of the research project, verbal consent was obtained from the patients. All the research procedures involving humans were consistent with the National Research Committee's ethical standards, the Helsinki Declaration of 1964, subsequent revisions, or equivalent ethical norms. Inclusion criteria for participation in the study encompassed providing informed consent and possessing a minimum level of reading and writing education. Exclusion criteria comprised the presence of psychological or neurological disorders and unwillingness to participate in the research.

### 2.2 Procedure

After obtaining permission from the developer of the demoralization questionnaire, a process of translation from English to Farsi was conducted using the backward–forward translation method (Beaton et al., 2000). Two bilingual experts proficient in both Farsi and English independently translated the questionnaire into Farsi. The two translations were then combined to create a consolidated version of the questionnaire. Subsequently, two other specialist translators translated the questionnaire from Farsi back into English. A comparison was made between the English translation and the original text, and the finalized version was emailed to the developer for verification of conceptual accuracy. Upon receiving feedback and making necessary revisions, the questionnaire was forwarded to eight professors specializing in psychology and psychiatry at Tehran University's Medical Sciences department. These experts evaluated the content validity of each item and subscale based on factors, such as simplicity, relevance, and clarity.

To assess the content validity ratio and content validity index, Lavache, Walter, and Basel methods were employed. In Lavache's method, the experts evaluated the necessity, usefulness, and redundancy of each item.

The Walter Basel method involved rating each item on a four‐point Likert scale to determine its level of simplicity, relevance, and clarity (Lawshe, 1975).

Following the content validity assessment, initially, consent was obtained from the patients, following which the questionnaires were distributed to individuals admitted to the cancer department at Imam Khomeini Hospital. Subsequently, participants were afforded the opportunity to duly complete the provided questionnaires, like demoralization, MUNSH happiness, and Beck's Depression scales.

### 2.3 Instruments

#### 2.3.1 MUNSH happiness

The MUNSH scale is crafted to assess levels of happiness. In light of the diverse perspectives on psychological well‐being and the plethora of scales developed for its measurement, Kozma and Stone devised a test in 1980 that underscores both the quantity and intensity of positive and negative emotions. Each emotional facet encompasses two dimensions: short‐term and long‐term. The short‐term dimension comprises 10 items representing the state aspect (positive and negative), whereas the long‐term dimension consists of 14 items related to the trait aspect (positive and negative). Within each positive and negative state aspect, there are 5 questions, and within each positive and negative trait aspect, there are 7 questions, resulting in a total of 24 questions on the scale. Responses to these questions are rated on a three‐point scale (yes = 2, no = 0, I don't know = 1). Each positive and negative state aspect has a potential score range of 0–10, whereas each positive and negative trait aspect has a range of 0–14. The overall scale score is computed using a specific formula based on positive and negative state and trait aspects. The questionnaire comprises five statements pertaining to positive feelings and seven statements regarding positive experiences. Scores from these two categories are summed, with the total scores of statements concerning negative feelings and experiences then subtracted from this sum. Higher raw scores indicate better psychological states. Cronbach's alpha coefficient for this instrument was reported as .71, indicating acceptable internal consistency (Mousavi et al., 2015).

#### 2.3.2 Beck's depression

The Beck Depression Questionnaire is comprised of 21 items, and the total score ranges from 0 to 63. This test is advantageous as it is valid for assessing the severity of depression in both clinical and nonclinical populations. Since its development, the Beck Depression test has undergone extensive psychometric evaluation, and its validity and reliability have been confirmed in numerous studies, including those conducted in Iran. The reliability of the Beck Depression Questionnaire has been demonstrated through various measures. Cronbach's alpha coefficient, indicating internal consistency, is reported as .89, whereas the correlation coefficient is .89. Additionally, the test–retest reliability coefficient is .94, with a 2‐week interval among administrations (Oliver et al., 2007). Furthermore, the questionnaire exhibits a high level of internal consistency, with a coefficient of .87, and the test–retest reliability coefficient is .73 (Ghassemzadeh et al., 2005). These findings support the reliability and stability of the Beck Depression Questionnaire in measuring depressive symptoms.

#### 2.3.3 Demoralization scale

The demoralization questionnaire, developed by Kissane et al. (2004), consists of 24 questions that assess 5 components. Respondents rate each item on a five‐point Likert scale, ranging from never (0) to always (4). The total score is obtained by summing the responses, with higher scores indicating higher levels of demoralization. The psychometric properties of this questionnaire have been reported to be satisfactory. The reliability of the demoralization questionnaire is supported by Cronbach's alpha coefficients for its five components. Specifically, the alpha coefficients for the loss of meaning and purpose, dysphoria, disheartenment, helplessness, and sense of failure components are reported as .87, .85, .89, .84, and .71, respectively (Kissane et al., 2004). These coefficients indicate good internal consistency and reliability of the questionnaire in assessing the various dimensions of demoralization.

### 2.4 Statistical method

To assess the validity of the questionnaire structure, EFA was conducted using SPSS 26 software. According to the recommended guidelines, the sample size for EFA should be approximately 6–10 times the number of items in the questionnaire (Hajizadeh & Asghari, 2011). In our study, the sample size exceeded the minimum requirement of six times the number of questionnaire items. Before conducting factor analysis, the adequacy of the sample size was evaluated through the Kaiser–Meyer–Olkin (KMO) measure and Bartlett's test of sphericity. The KMO value ranges from 0 to 1, with a value of 0.70 or higher indicating suitability for factor analysis. Bartlett's test assesses the correlation matrix among variables. Significance at the .05 level suggests a significant relationship among variables, indicating the potential to uncover a new factor structure from the data (Habibpor, 2011). Principal axis factoring and varimax rotation methods were employed in the EFA to identify distinct dimensions of demoralization. The decision regarding the number of factors was based on the eigenvalue criterion, where factors with eigenvalues greater than 1 were retained. Furthermore, a minimum factor loading of .40 was set as the threshold for item inclusion (Habibpor, 2011).

In CFA, the researcher aims to assess the validity of the factor structure of a set of observed variables (questions). The hypothesis being tested is whether there is a relationship between the observed variables (questions) and underlying latent variables (factors) (Kalantari, 2009). For CFA, the recommended sample size is between 5 and 20 participants per estimated parameter, which in our study is approximately 5 times the number of questionnaire items (Kalantari, 2009). To examine the suitability of the demoralization questionnaire structure in Iranian society, CFA was conducted using Amos 24 software. Fit indices were examined to evaluate model fit, including *χ* ^2^/*df* (chi‐square divided by degrees of freedom) ratio less than 3.0, comparative fit index (CFI) and non‐normed fit index greater than .9, root mean square error of approximation (RMSEA) less than .08, and standardized root‐mean‐square residual less than .05 (Wu, 2009). The reliability of the questionnaire was assessed using Cronbach's alpha coefficient, McDonald's omega, and Guttman's lambda coefficient tests. The acceptable range for Cronbach's alpha is .70–.94 (Taber, 2018), whereas omega internal consistency and Guttman's lambda‐6 are typically deemed acceptable when the estimate reaches .70 or above (Fumeaux et al., 2016; Viladrich et al., 2017). Convergent, divergent, and discriminant validities were examined by assessing the correlation among depression variables, such as Beck's Depression Inventory and the MUNSH happiness scale. Based on conducted studies, demoralization exhibits a correlation with depression, thereby establishing an inverse relationship with happiness (Rezaee et al., 2016; Tang et al., 2020).

## 3 RESULTS

The validity of the demoralization questionnaire was assessed using the content validity ratio (CVR) and content validity index (CVI). For CVR, a score higher than .85 and for CVI, a score higher than .75 are considered acceptable (Lawshe, 1975). In this study, the CVR scores ranged from .875 to 1, indicating good content validity. Similarly, the CVI scores ranged from .75 to 1, further confirming the content validity of the questionnaire.

### 3.1 Demographic features

The study comprised 160 Iranian cancer patients. The mean age of the participants was 54.68 years (SD = 11.35), with ages ranging from 25 to 82 years.

This cross‐sectional study was carried out using an available sampling method, and 160 Iranian cancer patients participated in the present study. As demonstrated in Table 1, the majority of the participants were women (*N* = 112, 70%), housewives (*N* = 98, 61.3%), married (*N* = 130, 81.3%), in age group over 50 years (*N* = 104, 65.0%), had primary education (*N* = 91, 56.9%), and had breast cancer (*N* = 43, 26.9%).

**TABLE 1:** Participant sociodemographic features.

		Frequency	Percentage (%)
Gender			
	Female	112	70.0
	Male	48	30.0
Age			
	18–30	2	1.3
	31–50	54	33.8
	Up to 50	104	65.0
Occupation			
	Unemployed	11	6.9
	Housewife	98	61.3
	Self employed	25	15.6
	Employee	6	3.8
	Teacher	5	3.1
	Retired	15	9.4
Marital status			
	Single	30	18.8
	Married	130	81.3
Type of cancer			
	Stomach	21	13.1
	Womb	4	2.5
	Liver	8	5
	Chest	43	26.9
	Intestine	35	21.9
	Lung	16	10
	Ovary	8	5
	Leg	5	3.1
	Lymphoma	9	5.6
	Eye	2	1.3
	Sarcoma	1	0.6
	Melanoma	3	1.9
	Brain	4	2.5
	Blood	1	0.6
Education			
	Primary	91	56.9
	Diploma	38	23.8
	Bachelor	26	16.3
	Master	5	3.1

**FIGURE 1:**
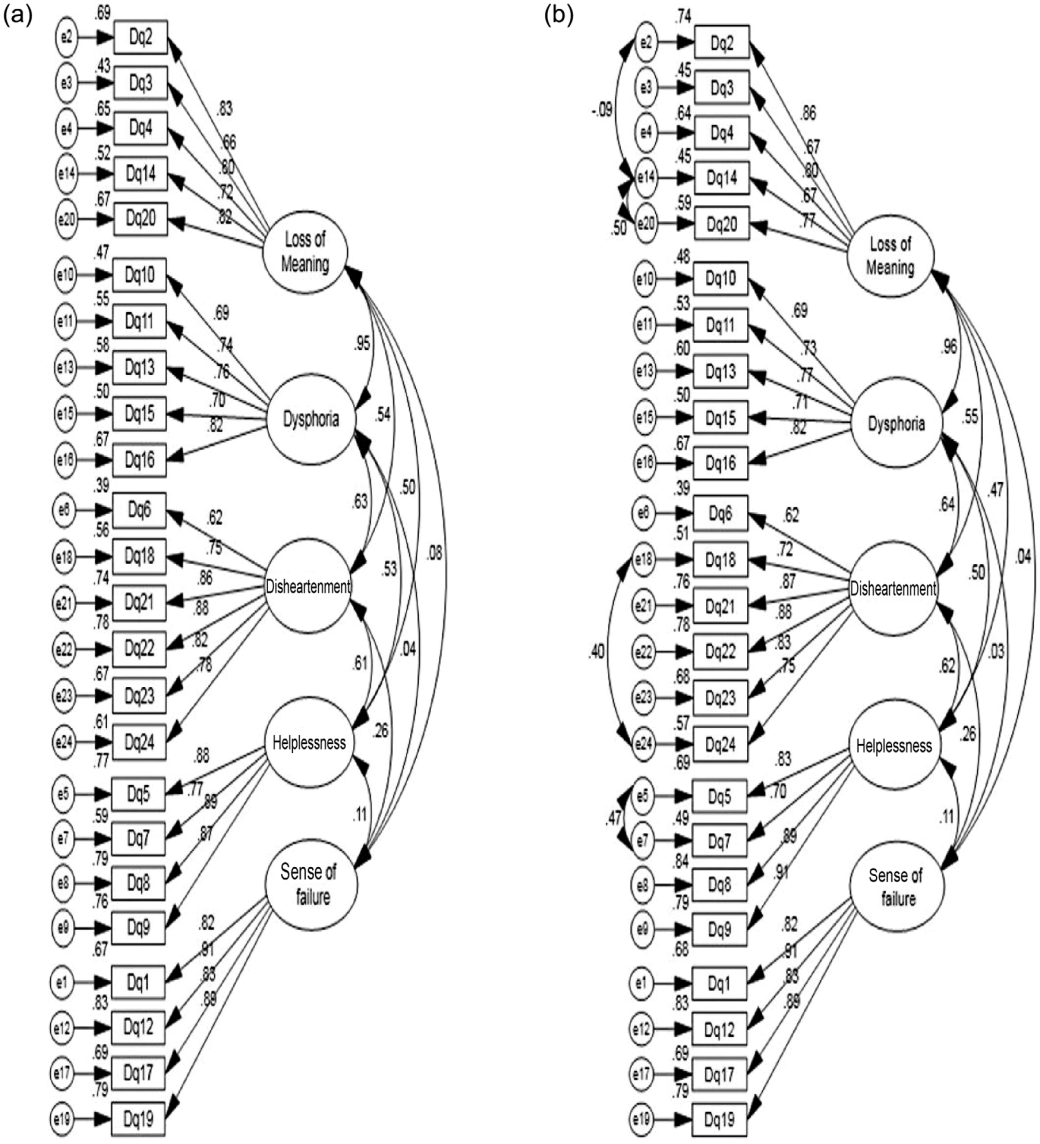
Model fit indexes of Demoralization Scale‐24 (DS‐24): (a) model fit indexes of DS‐II before modification; (b) model fit after modification.

Factor loadings of all DS's items were statistically significant (*p* < .001), and findings showed the standardized estimates for all items of demoralization were over .60 (Table 2). Investigating the fitness of the present model demonstrated that the model has a good fit with the data, and findings support the five‐factor model (Table 2 and Figure 1).

**TABLE 2:** Descriptive statistic indices for the items of the demoralization scale.

		Item's statistics			Item‐total statistics		
Items	Components	Mean	SD	F.L	V	I.T.	C.D.
Item1	Sense of failure	1.300	2.018	.82	.339	253.23	.921
Item2	Loss of meaning	1.195	.706	.86	.632	243.93	.915
Item3	Loss of meaning	1.204	.912	.67	.507	248.24	.917
Item4	Loss of meaning	1.073	.631	.80	.606	247.32	.916
Item5	Helplessness	1.010	.868	.83	.606	248.62	.916
Item6	Disheartenment	1.090	1.293	.62	.476	251.29	.918
Item7	Helplessness	1.179	.825	.70	.550	247.17	.916
Item8	Helplessness	1.124	.737	.89	.553	248.09	.916
Item9	Helplessness	1.079	.781	.91	.612	247.03	.915
Item10	Dysphoria	.879	.418	.69	.506	253.99	.917
Item11	Dysphoria	1.016	.812	.73	.597	248.78	.916
Item12	Sense of failure	1.223	1.918	.91	.348	253.88	.920
Item13	Dysphoria	1.354	1.050	.77	.594	242.09	.916
Item14	Loss of meaning	1.120	.662	.67	.579	247.29	.916
Item15	Dysphoria	1.243	.987	.71	.649	242.28	.915
Item16	Dysphoria	1.190	.937	.82	.690	241.97	.914
Item17	Sense of failure	1.291	1.82	.83	.181	259.77	.924
Item18	Disheartenment	1.145	1.406	.72	.588	246.51	.916
Item19	Sense of failure	1.400	2.162	.89	.306	253.34	.922
Item20	Loss of meaning	.887	.435	.77	.712	248.23	.914
Item21	Disheartenment	1.057	.775	.87	.714	244.24	.914
Item22	Disheartenment	1.067	.843	.88	.702	244.40	.914
Item23	Disheartenment	1.118	.806	.83	.708	243.02	.914
Item24	Disheartenment	1.238	1.250	.75	.602	244.12	.915
Total		25.06	16.39	–	–	–	–

Abbreviations: C.D., Cronbach's alpha if item deleted; F.L., factor loading; I.T., corrected item‐total correlations; V, scale variance if item deleted.

Table 2 shows the descriptive statistics of the 24 related items. The mean score obtained was 1.044, and all 24 items exhibited means in the 0.879–1.400 range. All corrected item‐total correlations were significant and surpassed .24 (see details in Table 2).

Based on the literature, a five‐factor model was tested through CFA, which provided marginal fit to data. The CFA findings for a five‐factor structure are illustrated in Table 1. The KMO coefficient was found to be 0.886, indicating the adequacy of the sample size for factor analysis. CFA displayed that the five‐factor structure provided a good fit to the data: CMIN/DF = 1.97 (*p* < 3); CFI = .92; IFI = .92; PNFI = .73; PCFI = .79; RMSEA = .078 (Table 3). These results showed all standardized factor loadings for all items were statistically significant (*p* < .01), supporting each item adequately. Factor loading for the DS‐24 items ranged from .67 to .91 (Table 2). As shown in Table 2 and Figure 1, all items of loads show a significant factor, and the standardized factor loading for all items is over .65.

**TABLE 3:** Confirmatory factor analysis (CFA) and model's fitness indexes.

	RMSEA (CI 90%)	CMIN/DF	CFI	IFI	PNFI	PCFI
Before modification	.091 (.081–.101)	2.32	.88	.89	.71	.77
After modification	.078 (.068–.089)	1.97	.92	.92	.73	.79

Abbreviations: CFI, comparative fit index; IFI, incremental fit index; PCFI, parsimony comparative fit index; PNFI, parsimony normed fit index; RMSEA, root mean square error of approximation.

### 3.2 Internal consistency reliability

Internal consistency reliability was investigated using data from the main study and based on the Cronbach's alpha, McDonald's omega, and Guttman's lambda coefficient test, in which Cronbach's alpha, McDonald's omega, and Guttman's lambda coefficient for the Persian Version of the DS‐24 were calculated .92, .91, and .84, respectively, which indicate good internal reliability (Table 4).

**TABLE 4:** Internal consistency reliability of the Demoralization Scale (DS‐24).

Variable	α	ω	*λ*6
Loss of meaning	.86	.86	.81
Dysphoria	.80	.86	.71
Disheartenment	.90	.90	.78
Helplessness	.91	.91	.86
Sense of failure	.92	.92	.78
Demoralization scale (DS‐24)	.92	.91	.84

Abbreviations: α, Cronbach's alpha; *λ*6, Guttman's lambda‐6; ω, McDonald's omega.

### 3.3 Convergent, divergent, and discriminant validity

The Pearson correlations acquired between the Persian Version of the DS‐24 with DS (DS‐II), MUNSH happiness, and Beck's Depression indicate good convergent validity (Table 5).

**TABLE 5:** Correlations among the Demoralization Scale‐24 (DS‐24), MUNSH happiness, and BDI‐21 with the DS‐II.

Variable	MUNSH happiness	Beck's Depression	Demoralization Scale‐II
Loss of meaning	−.231^**^	.309^**^	.565^**^
Dysphoria	−.164^*^	.185^*^	.568^**^
Disheartenment	−.335^**^	.274^**^	.662^**^
Helplessness	−.299^**^	.299^**^	.470^**^
Sense of failure	−.412^**^	.518^**^	.359^**^
Demoralization Scale (DS‐24)	−.405^**^	.442^**^	.712^**^

*p < 0.05, **p < 0.01, ***p < 0.001.

Table 5 demonstrates relationship among DS‐24 with other psychological variables in cancer patients; there was significant positive relationship between the total score of DS‐24 with Beck's Depression (*r* = .442, *p* < .01) and DS‐II (*r* = .712, *p* < .01). Moreover, there was significant negative relationship between the total score of DS‐24 with MUNSH happiness (*r* = −.405, *p* < .01). In other words, findings demonstrated good convergent and divergent validity for the Persian version of DS‐24 (see more details in Table 5).

## 4 DISCUSSION

The objective of this research was to undertake the translation and validation of the demoralization questionnaire among cancer patients within the Iranian societal context. The statistical findings substantiated that this questionnaire exhibits commendable measures of fit, validity, and reliability. This questionnaire has undergone translation and evaluation in various linguistic and cultural contexts across different nations (Cheng et al., 2019; Costantini et al., 2013; Mehnert et al., 2011; Mullane et al., 2009; Murri et al., 2020; Rudilla et al., 2016).

In this scholarly investigation, the outcomes of the exploratory statistical analysis revealed the presence of five factors like original study, the research conducted by Kissane et al. (2004) encompassed five‐factor components. Some of the research studies reported five factors, and another of them reported four components. For instance, the investigation of this questionnaire within the Irish community yielded five factors that exhibited a notable resemblance to the factors identified in the study conducted by Kissane et al. (2004) (Mullane et al., 2009); however, the findings of the study within German society indicated the presence of four distinct factors, namely, loss of meaning and purpose, disheartenment, dysphoria, and sense of failure. Notably, the factor of helplessness did not emerge as a separate factor in this context (Mehnert et al., 2011). Finally, the findings from Cheng et al.’s (2019) study conducted in China revealed the presence of four distinct factors. The extraction of four factors in certain societies in the mentioned studies, without specifying the helplessness factor as a distinct entity, may be attributed to the similarity of questions across factors. This potential oversight in translation may have overlooked subtle distinctions, possibly influenced by cultural differences.

The reliability coefficient for the entire questionnaire was documented as 0.920, and Cronbach's alpha for sense of failure, helplessness, disheartenment, dysphoria, and loss of meaning were, respectively, .92, .91, .90, .80, and .86. These findings collectively affirm the acceptable reliability of this questionnaire within the Iranian societal context. Cronbach's alpha coefficients for the total demoralization scores in various studies were as follows: in Kissane et al.’s (2004) study (.94), in Mullane et al. (2009), ranging from .72 to .93, in Costantini et al. (2013), the DS total score exhibited *α* = .90, in Mehnert et al. (2011), the total scale demonstrated *α* = .84, and in Cheng et al. (2019), a range of alpha coefficients from .720 to .894 were reported.

The outcomes of the concurrent validity and divergent analysis revealed a noteworthy positive correlation between demoralization and depression. Specifically, as an individual's depression score increased, their demoralization also exhibited a corresponding increase. Conversely, a negative and substantial correlation was observed between demoralization and happiness scores. In other words, an elevation in the happiness score among individuals experiencing demoralization was associated with a decrease in their demoralization.

Concurrent validity analysis and divergent analysis were conducted in various studies employing distinct questionnaires and ultimately demonstrated statistically significant findings. For instance, Kissane et al. employed the Beck Depression Questionnaire, McGill Quality of Life, Patient Health Questionnaire (PHQ), and Desire for a Hastened Death (SADH) in their concurrent validity analysis to assess their association with demoralization. Their investigation revealed relationships of statistical significance (Kissane et al., 2004). Rudilla et al. (2016), on the other hand, utilized the Depression and Anxiety Questionnaire in their study. Murri et al. (2020) incorporated the Patient Health Questionnaire‐9 (PHQ‐9), Brief‐Symptom Inventory‐18, Anxiety Subscale (BSI‐Anx), and EuroQol Group (EQ‐5D) in their analysis. Mehnert et al. (2011) examined depression and anxiety, coherence, and the existential vacuum (lack of meaning in life), revealing significant associations with the DS.

The limitation of this study pertained to the unequal representation of genders, as access to an equivalent percentage of women and men was hindered by the lesser willingness of ill men to participate in the research. Moreover, the study lacks representation of diverse socioeconomic backgrounds among cancer patients, potentially overlooking how economic status can influence demoralization experiences. And also, another limitation of this study is the absence of data regarding the various stages of cancer and the duration since cancer diagnosis, which may result in varying levels of demoralization, depression, and happiness.

## 5 CONCLUSION

In conclusion, it can be affirmed that cancer represents one of the most pernicious diseases, with profound repercussions on individuals’ psychological well‐being and overall quality of life. Demoralization often intensifies significantly in these cases. Consequently, the presence of a reliable instrument to assess demoralization in these patients assumes paramount significance in mitigating the progression of psychological distress. One of the highly effective tools for this purpose is the Kissane Demoralization Questionnaire, which has been translated and validated within the Iranian social context, yielding commendable results in terms of validity and reliability in this study.

Cancer often exerts a detrimental influence on the mental well‐being of patients, with demoralization being a prominent disorder frequently encountered. Given the questionnaire's demonstrated acceptable validity and reliability, it holds potential for future implementation in clinical settings. This instrument is proven to be instrumental in the timely detection of demoralization among cancer patients. We hope for this instrument to facilitate the timely administration of psychological and psychiatric interventions, thereby preventing the exacerbation of this condition.

## AUTHOR CONTRIBUTIONS

**Nazanin Mousavi**: Writing—original draft; writing—review and editing; supervision; project administration. **Mandana Piryaei**: Data curation. **Roghieh Nooripour**: Validation Add software and editing. **David Kissane**: Conceptualization; supervision. **Zahra Hooshyari**: Methodology. **Mohammad Effatpanah**: Supervision; project administration. **Nikzad Ghanbari**: Validation; software.

## CONFLICT OF INTEREST STATEMENT

The authors declare no conflicts of interest.

## FUNDING INFORMATION

This research received no specific grant from any funding agency in the public, commercial, or not‐for‐profit sectors.

### PEER REVIEW

The peer review history for this paper is available at https://publons.com/publon/10.1002/brb3.3589

## CONSENT TO PARTICIPATE

Before the implementation of the research project, consent was obtained from the patients.

## CONSENT FOR PUBLICATION

During the data collection, the names of the patients were not asked, and we used the code for data analysis.

## Data Availability

Data are available on request from the authors.
